# Different Educational Interventions on Individual Cognition of Garbage Classification Based on EEG Monitoring

**DOI:** 10.3390/ijerph19148567

**Published:** 2022-07-13

**Authors:** Rui Zhao, Xinyun Ren, Yan Liu, Yujun Li, Ruyin Long

**Affiliations:** 1 Faculty of Geoscience and Environmental Engineering, Southwest Jiaotong University, Chengdu 611756, China; ruizhaoswjtu@hotmail.com (R.Z.); rnxnyn@my.swjtu.edu.cn (X.R.); 2021300500@my.swjtu.edu.cn (Y.L.); 2 Department of Foreign Language and Culture, North Sichuan Medical College, Nanchong 637000, China; sammyleee@163.com; 3 School of Business, Jiangnan University, Wuxi 214122, China

**Keywords:** cognition, garbage classification, educational intervention, ERP, P300, LPP

## Abstract

Improvement in an individuals’ cognition is the key to promote garbage classification. This study takes university students as the research subjects, through three educational interventions, including the self-learning, heuristic learning, and interactive learning ways, to seek the most effective intervention based upon event-related potentials (ERPs) that is beneficial to enhance cognition of garbage classification. The results show that the experimental subjects induced P300 and LPP components, representing attentional changes and cognitive conflicts in classification judgments. There are differences in the amplitudes and peak latency of the two components corresponding to different interventions, indicating that the three educational interventions are able to improve the individual’s cognition level of garbage classification within a certain period of time. The interactive-learning intervention triggers the largest amplitudes of P300 and LPP, as well as the smallest peak latency, indicating its effect is the best. Such results provide insight into the design for an appropriate strategy in garbage classification education. The study also shows that an EEG signal can be used as the endogenous neural indicator to measure the performance of garbage classification under different educational interventions.

## 1. Introduction

Garbage classification is the premise of source reduction and resource utilization, which is of great significance to mitigate the pressure on terminal control. Thus, it helps to achieve sustainable management of municipal waste [1,2]. Governments have made great efforts in establishing infrastructure construction, policy incentives, and public education to encourage garbage classification [3,4]. But the actual effect has not yet met expectations. On the one hand, garbage classification-related behavior is a complex decision-making process, influenced by demographic characteristics such as gender, age, and education level [5]. On the other hand, the public has not fully recognized the environmental benefits brought by classification, resulting in significant differences between willingness and actual behavior [6]. In this context, education has become the key to enhancing public awareness, ensuring the implementation of garbage classification policies, and improving classification efficiency [7,8].

Different education ways may produce different intervention effects. The environmental education mode is generally based on a heuristic-learning model to encourage pro-environmental behaviors through instruction on environmental protection and sustainable development [9,10]. Hashimoto-Martell et al. (2012) and Rodríguez-Barreiro et al. (2013) analyzed the learning effects of the students who participated in courses related to environment and ecology, and found that case studies in these courses can significantly improve the students’ sense of learning [11,12]. Gan and Gal (2018) further proposed to optimize the curriculum by enhancing behavioral demonstration and guidance in the teaching to help students make positive contributions to the environment [13]. Syed-Abdullah (2020) found that students’ environmental awareness can be improved by linking environmental knowledge with the common sense of life [14]. Cordero et al. (2020) quantified the actual performance of environmental courses on pro-environmental behaviors. Through follow-up observations, they found that the average annual carbon emissions of the interviewed students decreased by 2.86 tons compared with those before education [15].

With the diversification of knowledge dissemination, the environmental education mode dominated by heuristic learning starts to transform into autonomous learning [16]. For example, self-learning platforms represented by massive open online courses (MOOCs) are able to deliver environmental knowledge without limitation by space and time [17,18]. Lin et al. (2015) believed that MOOC platforms have the advantage of diverse content in environmental education, which can help individuals better understand the information and improve their sense of learning [19]. Watson et al. (2016) proposed methods to improve the educational performance on MOOC platforms, especially for those who do not have clear learning goals or motivations, by embedding functions such as learning reflection, completion strategies, etc. into the platform to strengthen self-regulation during the learning process [20]. Kovanović et al. (2018) considered that MOOCs should not aim at transferring predetermined knowledge, but should encourage learners to build a knowledge system that can connect with their daily behavior [21]. Ma and Zhu (2021) evaluated the influence of various media on the formation of garbage sorting behavior, including television, mobile phones, the internet, etc. The results showed that more than half of the respondents believed the above-mentioned media may be beneficial to facilitate the formation of a classification habit [22]. Gu et al. (2020) and Angill-Williams and Davis (2021) found that direct, simple expressions, e.g., embedding typical environmental crises in public service advertisements, including water scarcity, global warming, etc., can help improve the individual’s self-learning effect and express a more pro-environmental attitude [23,24].

In order to enhance the penetration and feedback of education, a large number of studies have explored how the human–computer interaction (Web API, VR, etc.)-based environmental education scenarios may contribute to stimulating individuals’ subjective initiatives [25]. Ro et al. (2017) developed the Cool Choices game, which aims to guide individuals to recognize the environmental benefits of energy savings, and found that the testee who participated in the game reduced household electricity consumption in a short term (generally within 6 months) [26]. Nelson et al. (2020) found that by comparing the viewing experience of the same biodiversity conservation film in the ways of virtual reality (VR) and ordinary 2D, the former helps to increase the individual’s perception of conservation issues [27]. Vuong et al. (2021) and Breves and Schramm (2021) tried to analyze the reasons why immersive media is more effective than ordinary media for environmental education, and found that the former can prompt participants to generate more learning attention and form a deeper memory, ultimately arousing their environmental awareness [28,29].

This study mainly focuses on discriminating the possible cognitive intervention effects on garbage classification, to seek for the best educational intervention to improve cognition of garbage classification. University students are taken as the experimental subjects because of their relatively high education level and ability to understand knowledge, and because they can be regarded as pioneers for promoting garbage classification and positively influencing other groups [30]. The results can be used to guide the formulation of education programs and provide insight into the policy implications for garbage classification.

## 2. Event-Related Potentials Related to the Cognitive Process

When examining the effect of education on garbage classification, existing studies mainly explained motivation by using the theoretical models of planned behavior, group behavior, and norm-activation based on questionnaires, focus groups, and in-depth interviews [31,32,33]. However, the above methods are difficult to dynamically measure the changes in individuals’ cognition of garbage classification. Neuroscience can fill such a gap by using brain imaging techniques, e.g., taking event-related potentials (ERPs) to reflect the individual’s neural activity caused by specific time stimulations [34,35].

Specifically, studies generally explain a specific cognitive process by analyzing the brain states represented by typical components in the ERPs [36]. Among them, the late positive potential (LPP) is generally used to assess attentional conflict caused by external stimulus-driven or subjective goal orientation, and higher LPP amplitudes represent larger cognitive conflicts [37,38]. Crites and Cacioppo (1996) first discovered that this process triggers LPP components when they studied the process of food categorization [39]. Cunningham et al. (2005) also observed LPP components when individuals evaluated a social event [40]. Brattico et al. (2010) took music preference as a typical case, and found that the favored judgment triggers LPP [41]. Sun et al. (2017) further confirmed that LPP can be used to discriminate deterministic and ambiguous evaluations, and the latter tends to induce larger LPP magnitudes [42]. Abid et al. (2021) assessed attention allocation to emotional stimuli by using LPP as a tool [43]. Similarly, Wang et al. (2022) took LPP as an indicator to explore the financial credit decisions of small and medium-sized enterprises [44].

Compared with LPP, P300 is inclined to reveal the cognitive function of an individual. Specifically, when an individual completes a task, the latency of P300 corresponds to the time taken to choose an option, and the amplitude of P300 reflects the difficulty of the option [45,46]. Cui et al. (2017) found that when the individual’s cognitive ability declines, the latency of P300 is prolonged and the corresponding amplitude becomes smaller [47]. Dickson and Wicha (2019) further verified such a phenomenon when individuals performed calculations; that is, when subjects chose different calculation methods, the performance of P300 was quite different [46]. Particularly, the P300 amplitude was smaller, the latency was longer, and the LPP amplitude was larger compared with the correct calculation method. Mijović et al. (2019) examined the impact of attentional changes on an individual’s assembly operations, and found that the P300 amplitude increases with the complexity of the operation [48]. Alicart et al. (2020) identified that gossip information was more deeply remembered than trivia, and the related P300 amplitude was larger, indicating that it was positively related to the attention state [49]. Cardoso et al. (2021) tried to distinguish the cognitive processes expressed by P300 and LPP, in which the former was associated with concentration, whereas the latter was more closely related to emotional changes [50]. Liu et al. (2022) used P300 to explore the impact of social norms on the willingness to use recycled water. The results found that when an individual’s willingness is lower than social norms, there will be a larger feedback-related negative amplitude [51].

This study follows the above ideas, and introduces ERPs into the evaluation of educational intervention on individual’s garbage classification. Through the analysis of the LPP and P300 components of the subjects, the study seeks to expand the traditional interpretation of these components by interaction with response accuracy to characterize the effect of educational intervention. By comparing the cognitive level with the characteristics of the measurable components, it is possible to explore the mechanisms underlying different educational interventions from a neuroscience perspective.

## 3. Materials and Methods

### 3.1. Experimental Design

**Figure 1:**
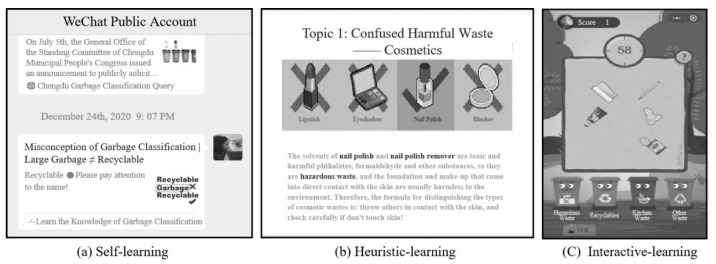
Instruction materials for different intervention groups. (**a**) Self-learning, (**b**) Heuristic-learning, (**c**) Interactive-learning.

Based on the social constructivist learning theory, learners can use the help of others or use various tools and information resources to achieve their learning goals [52]. This study designed three intervention ways, which are divided into self-learning, heuristic-learning, and interactive-learning ways. The learning materials corresponding to the intervention groups are shown in Figure 1.

Among them, the self-learning way was based on an instant messaging platform (WeChat Public Account) that posted the garbage classification-related information, shown in Figure 1a. For instance, the information contains what categories can be defined as recyclables, hazardous, etc. By using such media, the subjects receive messages flexibly and learn in more detail according to their time schedule. The heuristic-learning intervention in this study was mainly based on the instruction of a lecturer, which is a two-way communication, as shown in Figure 1b. The lecturer illustrated different garbage categories by using PPT slides embedded with figures and videos, as well as gave typical examples to help the subjects discriminate the easily confused categories. The subjects could propose questions when they had any query, to aid themselves in building a knowledge system regarding garbage classification. The lecturer and the subjects during the experimental period were the same people to avoid deviations in information transmission and reception due to individual differences. Interactive learning mainly depended upon the game form, as shown in Figure 1c, such as prize competitions and simulation games to arouse the subjects’ learning interests regarding garbage classification.

Before the experiment, a questionnaire survey on garbage classification cognition was distributed among the students within a university in Chengdu City, China. According to the answering accuracy, 25 students with similar knowledge levels were selected as paid subjects to participate in the experiment voluntarily. The subjects included 11 female students and 14 male students, aged between 18 and 20, with an average age of 19.5. They were randomly divided into 4 groups, including experimental group 1 (6 students for self-learning), experimental group 2 (6 students for interactive learning), experimental group 3 (6 students for heuristic learning), and the blank control group (7 students). The experiment was approved by the Ethics Committee of Southwest Jiaotong University (approval number: SWJTU-2103-037). Each subject was informed in detail about the specific process of the EEG experiment, and signed the informed consent form, which indicated that the experiment would not cause any adverse physical and mental harm. After the experiment was completed, each participant was given a reward. All the subjects were required to be right-handed, with normal or corrected-to-normal vision, and no neurological or psychiatric history.

**Figure 2:**
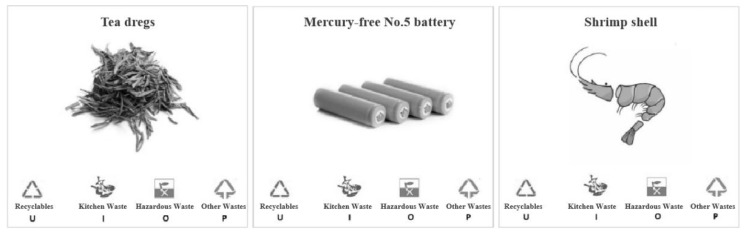
Examples of stimulus materials (Stimulus material is presented in figures, upper part is the garbage name, middle part is the garbage example, lower part is the options of garbage categories. Keystrokes of U/I/O/P).

In the experiment, 160 pictures of garbage that are common in daily life were randomly selected as the stimulus and were processed uniformly to ensure that the color and brightness are similar, and the size is uniform, as shown in Figure 2. The experiment required the subjects to judge the category of garbage by keystroking U/I/O/P according to the figure displayed in the center of the computer monitor. According to the experimental results, the answering accuracy and response time were extracted to discriminate among the subjects whether there was a difference before and after the intervention, as well as whether there was a difference among the intervention groups. The EEG data was superposed and averaged according to the code signs transmitted by the E-prime program to obtain the corresponding ERP components to further compare whether there were differences in neural activity before and after the intervention, and thus to explore the best intervention way.

### 3.2. Experimental Device

The experiment employed a portable EEG acquisition device, developed by NeuroSky, using dry electrode sensors, which are non-invasive to the human body. A number of studies have applied NeuroSky to medical diagnostics. For instance, Chu et al. (2020) and Serrano-Barroso et al. (2021) assisted physicians in screening patients with attention deficits measured by NeuroSky [53,54]. Khan et al. (2021) used NeuroSky as a brain–machine interface to convert the EEG signals it received into commands to drive wheelchairs, helping people with motor dysfunction to improve their quality of life [55]. Such a simple device is able to provide a stable recording function, and has sufficient data quality compared with the conventional laboratory equipment [56,57].

The recording electrode of the acquisition system was placed on Fp1 (left prefrontal cortex, only in contact with the skin), and the reference electrode was clamped on the left earlobe. The ERP components, including P300 and LPP, observed at FP1 were highlighted by the significant changes during the cognitive improvement [49]. The sampling frequency was 512 Hz, and data recording was realized by connection to a laptop via Bluetooth.

The experimental task was presented by a laptop monitor. The electroencephalogram was recorded using NeuroSkyLab, by which EEG was continuously recorded and taken offline for analysis by using EEGLAB. The analysis time was set as 1000 ms, from 200 ms before the stimulation to 800 ms after the stimulation. Kappenman and Luck (2010) found that the impedance-dependent attenuation of P300 was minimized when using a 0.5 Hz high-pass filter [58]. Thus, this study chose a high-pass filtering rate of 0.5 Hz and a low-pass filtering rate of 30 Hz. A 58–62 Hz trap filter was used to reduce the 60 Hz line noise, and the abrupt voltage offset was removed artificially. An independent component analysis (ICA) of the ephemeral metadata was performed by using the “Infomax” method embedded in the “runica()” function of EEGLAB [59]. Such analysis is powerful to identify and remove components that cause large artifacts (e.g., blinks, lateral eye movements, muscle tone, etc.) [60].

### 3.3. Experimental Procedure

The experiment had five sessions. In fact, the subjects were asked to participate in 5 sessions of the experiment, including 1 session before the intervention (T0) and 4 sessions during the four-week intervention (T1–T4). Each session was consisted of 24 trials, with a total of 120 trials per subject. Furthermore, 25 subjects in 4 groups participated in a total of 3000 trials in 5 sessions (e.g., 25 × 5 × 24). Each trial was presented with one picture, by which the subject was given a total of 120 pictures as the stimulus. These 120 pictures were randomly selected and not repeated among the 160 images.

**Figure 3:**
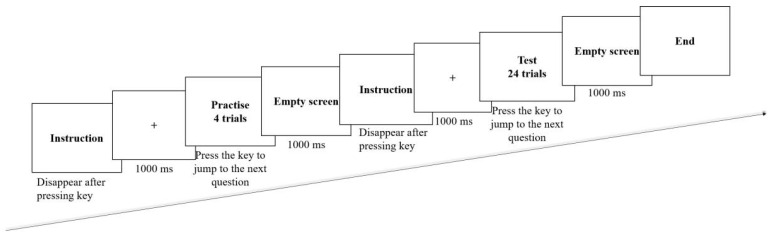
The stimulus presentation designed in the experiment.

The experiment was programmed by the E-Prime 2.0 software to present the stimulus, and recorded all the data related to the subjects’ keystrokes. Before the experiment, the subjects were asked to read the experimental instructions. The presentation sequence of an experiment was as follows (See Figure 3): First, the fixation-cross was presented in the center of the computer screen for 1000 ms, followed by a stimulus figure for 5000 ms. Compared with the normal stimulus duration of 1000 ms, the stimulus duration set to 5000 ms in this study not only gave sufficient time for EEG sampling, but also took possible behavioral responses (e.g., keystroke) into account. For example, in Herbes et al.’s (2015) study on consumers’ willingness to pay for green electricity products, subjects were asked to keystroke to decide whether the pricing was in line with their expectations. The keystroking time per stimulus was set to 1200 ms on average, with the longest reaching 1600 ms in their study [61]. In addition, the keystroke is a hand-generated movement that requires time to return to the initial state. Second, the subjects were required to make judgments based on the stimulus. Once the keystroke was completed, a blank screen with a duration of 500 ms appeared. In addition, this study also provided practice trials to help the subjects familiarize with the operating procedures and keystrokes. The related results were not included in the analysis.

### 3.4. Experimental Measurement

For the behavioral data analysis, this study mainly examined the response time and response accuracy of the subjects. The former refers to the time from the subject seeing the stimulus figure to making a keystroke response, which represents the shortest time for the subject to make a classification judgment after cognitive processing. The accuracy of answering questions refers to the ratio of the number of correct classification judgements completed by the subjects to the total number of experiments. The subjects who did not respond within the specified time were automatically regarded as incorrect responses.

With regard to the EEG monitoring, this experiment mainly investigated two positive and late components, P300 and LPP. P300 is the component that peaks between 300–500 ms after the onset of stimulation, and is also the main event-related potential that characterizes the degree of individual concentration [40,62]. The higher the individual’s sustained attention, the greater the P300 amplitude. The amplitude of LPP that emerges after P300 represents the level of cognitive conflict [63]. The greater the magnitude of the evoked amplitude, the higher the uncertainty of the individual’s judgment on the classification. In this study, the time window corresponding to the two components was selected to extract the corresponding peak value and latency. The group was used as the between-subject variable, and the intervention stage as the within-subject variable, to perform the repeated measures of variance test. The EEGs formed by all the trials were added to the ERP calculation. For the different groups and time sessions, each ERP calculation was based on 144 trials (6 × 24).

The experimental results are presented from the analysis of behavioral data, including the statistical analysis of the response accuracy under different interventions in the four sessions. Next is the analysis of the ERP components, performed to investigate the characteristics of the P300 peak latency and LPP amplitude under different interventions in the four sessions. The behavioral data analysis was performed by using a *t*-test. With regard to the EEG data, the repeated measures of variance test was used in this study, and the sphericity test was performed to determine whether there is a correlation between the data of each session. If there is a correlation, it is necessary to correct the degrees of freedom before performing the analysis of variance. Otherwise, performing the analysis of variance directly may increase the probability of a type I error [64].

## 4. Results

### 4.1. Behavioral Analysis

#### 4.1.1. Response Accuracy

Without intervention, the difference in accuracy between the control group and the self-learning group (*t* = −0.074, *p* = 0.942), the control group and the heuristic-learning group (*t* = 0.160, *p* = 0.876), and the control group and the interactive-learning group (*t* = 0.420, *p* = 0.682) did not reach the level of significance. The difference in response time between the control group and the self-learning group (*t* = 1.125, *p* = 0.285), the control group and the heuristic-learning group (*t* = −0.641, *p* = 0.535), and the control group and the interactive-learning group (*t* = 1.299, *p* = 0.221) was also not significant, indicating the homogeneity and rationality of the grouping.

**Figure 4:**
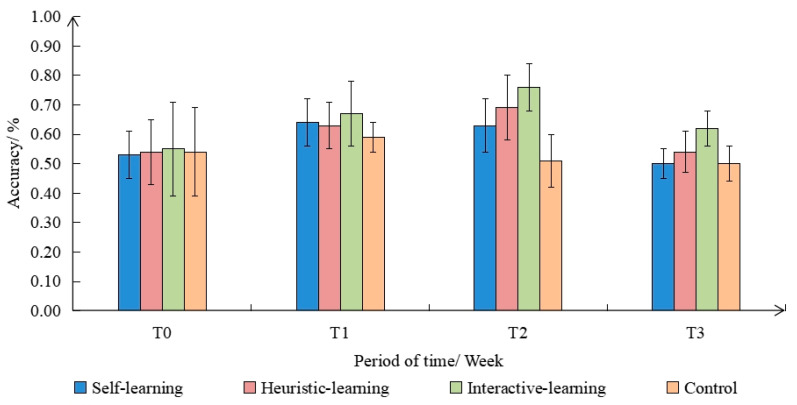
Changes in the accuracy of different groups and time periods.

Through the statistical analysis of the classification accuracy under different interventions, it was found that the accuracy shows a trend of rising first and then decreasing, especially in the T3 stage, where the accuracy presents a significant decline, as shown in Figure 4. In addition, with the group as the between-subject variable and the test time as the within-subject variable, the repeated measures analysis of variance was performed on the classification accuracy. The results of the sphericity test showed that *p* > 0.050, which met the Huynh–Feldt condition, and the time factor within the subject was statistically significant (F(3,9) = 17.260, *p* < 0.001), indicating that there were significant differences in the classification accuracies under different intervention sessions. There was a significant difference between the groups (F(3,9) = 7.677, *p* < 0.001). The average accuracy of the interactive-learning group was significantly higher than that of the other groups. There were significant differences between the interactive-learning group, the heuristic-learning group, and the control group, whereas the difference between the self-learning group and the control group was not significant. The accuracy of the three intervention groups was significantly higher in T1 and T2 than T0, but there was no significant difference between T3 and T0.

#### 4.1.2. Response Time

**Figure 5:**
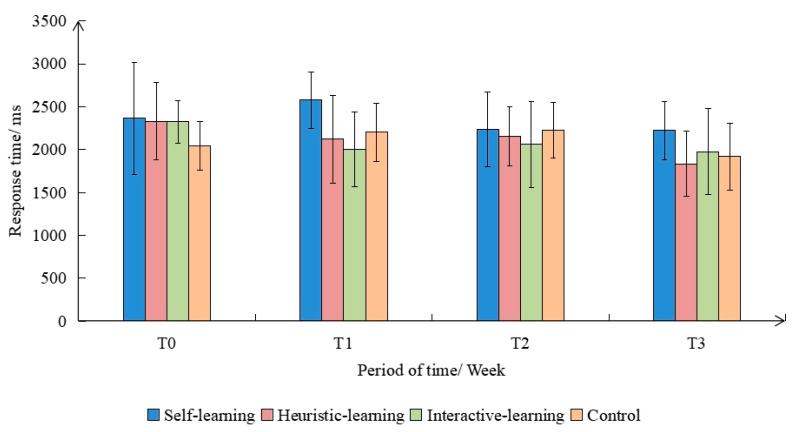
Variation of response time in different groups.

With the group as the between-subject variable and the response time as the within-subject variable, the repeated measures analysis of variance was performed on the response time. The sphericity test results showed that *p* > 0.050, which satisfied the Huynh–Feldt condition, indicating that the response time was statistically significant (F(3,9) = 3.400, *p* < 0.05). There were significant differences in the response times corresponding to different sessions, and the difference between groups was not significant (F(3,9) = 1.889, *p* < 0.05). However, the interaction-learning group had the shortest response time, and the self-learning group had the longest response time, as shown in Figure 5. There were significant differences between T1, T2, and T3 within the group.

### 4.2. ERP Analysis

#### 4.2.1. P300

**Figure 6:**
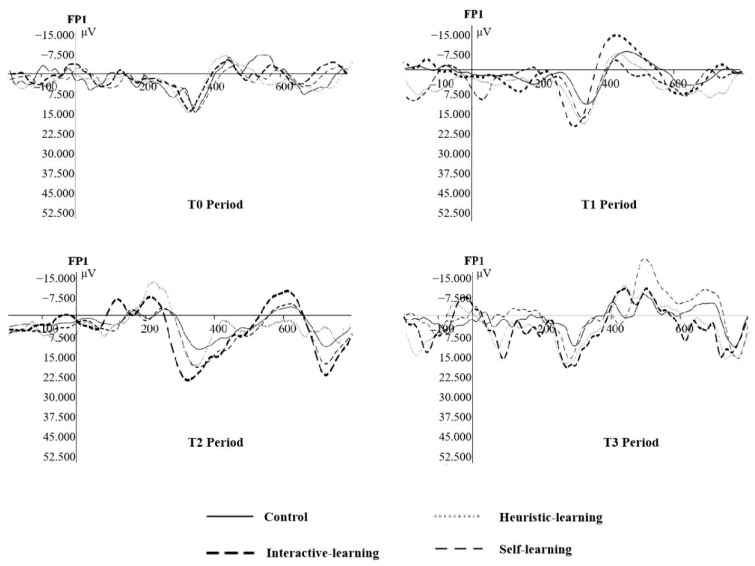
ERP induced by the classification judgement.

The EEG data in this experiment were segmented from 200 ms before stimulation to 800 ms after stimulation. Taking 200 ms before stimulation as the baseline, an average superposition was performed to analyze the changes of EEG signals in different groups before and after intervention, as well as in each intervention group. In order to show the waveforms and amplitudes of the components under different interventions, the brain waves of the subjects on the FP1 electrode were drawn, as shown in Figure 6.

**Figure 7:**
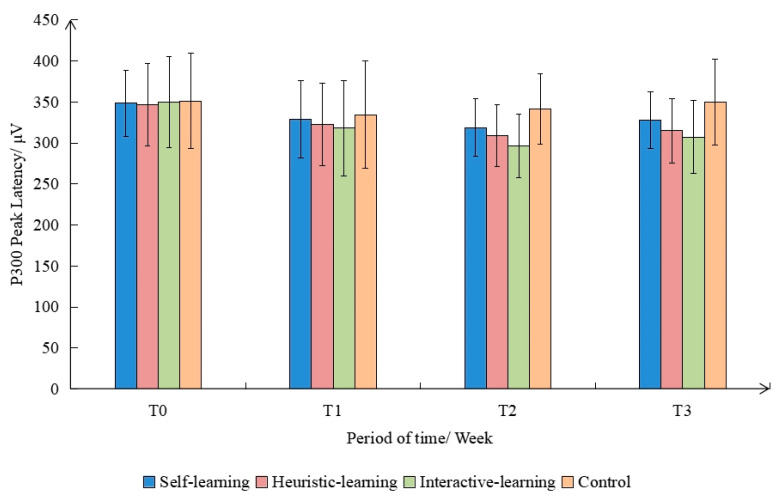
Comparison of P300 peak latency in different groups and time periods.

According to the relevant studies on the composition of P300, the peak of P300 appears at about 300 ms [65]. This study selected 250 ms to 380 ms as the time window for P300 component analysis, in terms of the experimental results. Figure 7 shows the P300 peak latency of subjects in different groups. It was found that the main effect between groups was significant (F(3,9) = 34.283, *p* < 0.001, η^2^ = 0.873). The latency period of the control group was significantly greater than that of each intervention group (*p* < 0.01), and the peak latency period of the interactive-learning group was the smallest. The main effect within the tested group was significant (F(3,9) = 21.288, *p* < 0.001, η^2^ = 0.810). The peak latency of subjects before the intervention was significantly greater than that after the intervention (*p* < 0.05). The above results indicate that the group-time interaction had no significant effect on P300 peak latency. After being educated, the subjects’ cognition of garbage classification was significantly improved, resulting in further improvement in the working memory to identify different garbage categories [66,67]. The specific performance shows that the response time for the classification judgement is reduced, and the intervention of interactive learning has the best effect.

**Figure 8:**
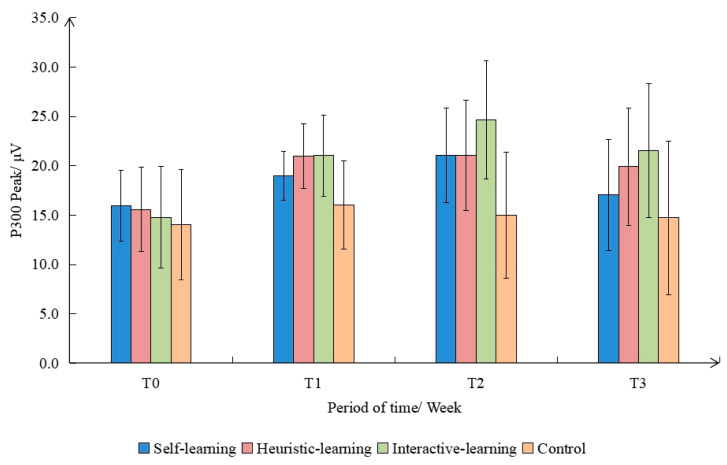
Comparison of P300 peaks in different groups and time periods.

Figure 8 shows the comparison of the P300 peak values corresponding to different groups of subjects. The main effect between subjects was significant (F(3,9) = 32.090, *p* < 0.010, η^2^ = 0.865). There were significant differences between the groups (*p* < 0.050), and the peak value of the interactive-learning group was greater than that of the other groups. The main effect within the experimental groups was significant (F(3,9) = 24.560, *p* < 0.001, η^2^ = 0.831). There was a significant difference between the peak in the T0 stage before the intervention and after the intervention. After the intervention, the difference between T2, T1, and T3 was significant. The interaction between group and intervention time was characterized as significant (F(3,9) = 9.439, *p* < 0.010, η^2^ = 0.654). The above results show that the interactive-learning group pays more attention to the classification judgment.

#### 4.2.2. LPP

**Figure 9:**
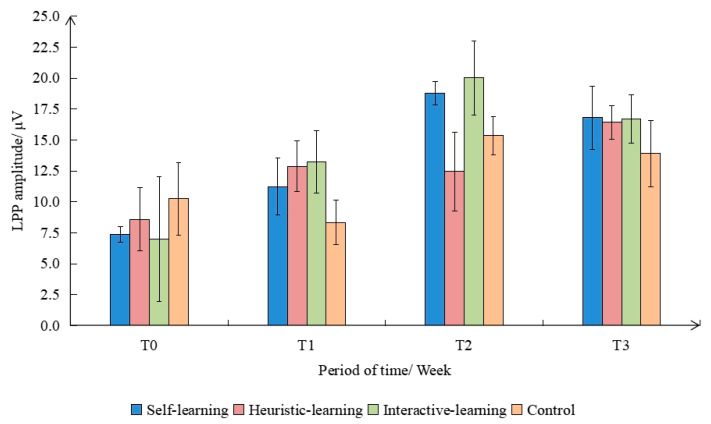
Mean and standard deviation of LPP amplitude under different interventions.

This study selected the segment between 550 ms and 750 ms after stimulus presentation as the time window for LPP component analysis. The EEG amplitudes within this time window were extracted. The LPP amplitude and standard deviation of the four groups under different interventions are shown in Figure 9, among which the LPP component caused by the interactive-learning group was the strongest, and the one caused by the control group was the weakest. There were significant differences in the amplitudes of LPP caused by different interventions, and the amplitudes were as follows: interactive-learning group > self-learning group > heuristic-learning group > control group.

The repeated measures of variance test for LPP amplitudes within the time window revealed a significant main effect between groups (F(3,9) = 3.419, *p* < 0.050). There was a significant difference in LPP amplitude between the interactive-learning group and the control group (*p* < 0.050), but the difference in the other groups was not significant. The effect within the group was significant (F(3,9) = 32.321, *p* < 0.001). There were significant differences in T1, T2, and T3 after intervention and the T0 stage before intervention, and T2 and T3 after intervention were significantly larger than the T1 stage. In addition, the interaction effect of group and test time was also significant (F(3,9) = 8.235, *p* < 0.001). In the control group, different intervention times had no significant effect on peak value. However, different intervention times had a significant impact on the peak value in the intervention group. In the interactive-learning group and the self-learning group, there were significant differences between T3 and T4 and between T1 and T2. In the heuristic-learning group, T4 was significantly different from T1 and T3.

## 5. Discussion

This study combines the behavioral data and EEG data for discussion. With regard to the behavioral data, there were significant differences in the response accuracy and response time of the subjects with different interventions. After the interventions, the overall response accuracy of the subjects was significantly improved, and the response time was significantly reduced. However, after the intervention of the T3 period, the accuracy dropped significantly. This may be due to the continued decline of the subjects’ interest in learning due to the continuous intervention. When individuals have no interest in learning or lack motivation, they will feel bored, have a negative psychological state, and form learning burnout [68]. In order to achieve the best intervention effect, the duration of the intervention is particularly important. This study thus suggests the intervention duration should be controlled within 14 days.

The repeated measures of variance test results showed that there were significant differences among groups. There were significant differences in the response accuracy and response time between the intervention group and the control group, and the difference in the response accuracy between the interactive-learning group and the control group was the largest. There were significant differences in the response accuracy at each intervention time period. The higher the response accuracy, the shorter the response time of the subjects, indicating that the intervention played a key role.

From the perspective of the EEG data, different interventions induced the P300 and LPP components when the subject made judgements on garbage classification. The P300 peak and LPP amplitudes induced by the interactive-learning group were the largest, and the P300 latency was the shortest. The shorter P300 latency indicates that the subjects can adjust their behaviors according to the experimental results, which prompts the subjects to make correct classification decisions [42]. This also shows that the instruction of garbage classification needs to provide timely feedback in order to improve the learning effect and improve cognition of classification. After the intervention, there were significant differences in the P300 peak values corresponding to the self-learning group, the heuristic-learning group, the interactive-learning group, and the control group. Especially notable was that the amplitude of the interactive-learning group became significantly larger. Related studies have shown that attention affects P300, and stimulus materials with a higher similarity are more likely to capture more attentions, thus causing larger amplitudes [65]. In this experiment, when the stimulus was presented, the subjects quickly associated it with the knowledge that they have mastered. The greater the degree of matching between the stimulus and the knowledge, the greater the amplitude of the P300, which led to the individuals producing more attentions [69]. In addition, the interactive-learning group evoked a larger LPP amplitude compared with the other three groups, indicating that the subjects in the interactive-learning group showed a stronger reaction and maintained close attention to the stimulus.

The behavioral and EEG results confirmed that education had a positive effect on the improvement of cognition of garbage classification. Compared with self-learning and heuristic learning, interactive learning can effectively improve the efficiency of garbage classification. Such postulation regarding the performance of game-based interactive learning can be verified by similar studies. Papamichael et al. (2022) took the interactive design of a garbage classification game as an example, and pointed out that the indicators in the game (such as waste category, recycling possibility, etc.) can help players better understand the methods of garbage classification [70]. Lu et al. (2022) believed that educational games, compared with other educational methods, can better simulate the decision-making process and help individuals mobilize cognitive abilities in order to produce quick responses [71]. Cook et al. (2022) developed a game entitled “Cranky Uncle” to prevent the negative impacts of misleading information related to climate change, demonstrating through case studies that it fosters critical thinking on the discrimination of misinformation [72].

However, the study has certain disadvantages, which means there is room for further improvements. First, the interventions involved in this study may not necessarily be effective with a large group of subjects due to sample size and sample homogeneity. Although the sample size was small, it was sufficient in measuring the performance of the interventions. A large number of existing studies are also based on small samples (less than 30) to address the impact of stimuli on a subject’s cognitive processes through a statistical analysis of ERP components, which is similar to the aim of our study. For example, Wu et al. (2021) used 22 subjects as a sample to study the effects of different color stimuli on attention, and explained the effects of color stimuli on visual search, attention capture, and attention orientation with P2, N2b, and P3a, respectively [73]. Li et al. (2022) explored the neural processes induced by the central visual area when encountering danger, through an observation of the N100 and P200 related to 26 car drivers when they saw a traffic accident [74].

Second, the study only monitored the changes of event-related potentials at the Fp1 electrode, which may not fully highlight the intervention effect due to the measurement limitations of the experimental equipment. However, the study aimed to explore whether the educational performances produced by different interventions are distinct, indicated by the classification effects of different groups being statistically significantly different. ERP components, including P300 and LPP, observed at Fp1 are highlighted by changes during the cognitive improvement [49]. P300 represents attentional resource allocation during memory updating [75,76], which also reflects the activity of the left temporoparietal brain region and the left prefrontal area [77,78,79,80]. Begum and Reza (2021) used a 128-electrode sensor net to study auditory cognitive function in pregnant women, and found that the pregnancy group produced the highest amplitude at the Fp1 electrode in terms of the P300 component [81]. The P300 component measured in the Fp1 position indicates that the attribution of the individual is similar to the changes in the neural processes caused by the cognition of garbage classification.

For LPP, we acknowledge that the six centroparietal sites with the most significant changes have not been selected: CP1, CP2, CPz, P1, P2, and Pz [82,83], due to the restrictions by the experimental device. However, LPP is caused by the reciprocal activation of frontal and occipital-parietal regions, which is close to Fp1 [84,85]. It is worth noting that the LPP components measured at the Fp1 position have good expression ability in tasks such as comparison, selection, and judgment [86,87]. For instance, Ding et al. (2021) selected fourteen conductive electrodes (Fp1, FP2, F3, FZ, F4, C3, CZ, C4, P3, PZ, P4, O1, LZ, and O2) to indicate the emotional changes caused by product color, by which significant LPP components were generated at the Fp1 and P4 positions. The high color consistency of the product was favorable for inducing a larger LPP amplitude [88].

Future studies will need to consider expanding the sample size, and monitoring and comparing with other potential changes to explore the appropriate environmental education measures applicable to different groups.

## 6. Conclusions

This study investigated three educational interventions on university students’ cognition of garbage classification, including self-learning, heuristic-learning, and interactive-learning ways, to explore the most effective intervention for improving the efficiency of garbage classification. Studies have shown that different interventions have induced P300 and LPP components that characterize classification. After the intervention, the response accuracy was significantly improved, and the response time was also significantly shortened, indicating that the three educational interventions have a positive impact on the improvement of cognition of garbage classification. Compared with the other groups, the interactive-learning group induced larger amplitudes of P300 and LPP, and had the highest accuracy and shortest response time, indicating it had the best intervention effect. The classification accuracy in each group showed a certain decrease after three weeks of education, which led to the inference that long-term intervention may lead to a decline in learning interest. Therefore, it is suggested that the time for educational intervention should be set within 14 days.

## Data Availability

The data are available by reasonable request to the corresponding author.
